# Enhancing network traffic detection via interpolation augmentation and contrastive learning

**DOI:** 10.1371/journal.pone.0338546

**Published:** 2025-12-22

**Authors:** Lei Li, Qiang Zhou, Xinlong Yang, Linye Chen

**Affiliations:** Ningbo University, College of Science and Technology, Ningbo, Zhejiang, China; American International University Kuwait, KUWAIT

## Abstract

With the rapid advancement of information technology, the Internet, as the core infrastructure for global information exchange, faces increasingly severe security challenges. However, traditional network traffic detection methods typically focus solely on the local features of traffic, failing to comprehensively consider the global relationships between traffic flows. This limitation results in poor detection performance against multi-flow coordinated attacks. Additionally, the inherent imbalance in real-world network traffic data significantly hampers the performance of most models in practical scenarios. To address these issues, this paper proposes a network traffic detection method based on data interpolation and contrastive learning (TICL). The method employs data interpolation techniques to generate negative samples, effectively mitigating the data imbalance problem in real-world scenarios. Furthermore, to enhance the model’s generalization capability, contrastive learning is introduced to capture the differences between positive and negative samples, thereby improving detection performance. Experimental results on two publicly available real-world datasets demonstrate that TICL significantly outperforms existing intrusion detection methods in large-scale data scenarios, showcasing its strong potential for practical applications.

## 1 Introduction

With the rapid advancement of information technology, High-Performance Computing (HPC) systems have become the core infrastructure in critical fields such as scientific research, engineering simulations, and financial analysis. These systems, renowned for their robust data processing capabilities and ability to execute highly complex computational tasks, serve as critical infrastructure driving innovation and progress across industries. However, as the computational power and scale of HPC systems continue to expand, they are increasingly exposed to sophisticated security threats. The inherently open and interconnected nature of HPC environments makes them attractive targets for cyber attackers, particularly those aiming to compromise sensitive data or disrupt critical operations. Consequently, addressing the evolving threat landscape and implementing robust defensive mechanisms have emerged as paramount priorities to ensure the secure and reliable operation of HPC systems.

To protect HPC systems and their users, it is crucial to promptly identify and mitigate intrusive activities and rapidly deploy defensive measures. Traditional security solutions, often designed for conventional IT environments, are typically ill-equipped to handle the unique complexity and scale of HPC infrastructure. However, Network Intrusion Detection Systems (NIDS) offer a promising solution by continuously monitoring network traffic within HPC systems, serving as a core line of defense against unauthorized access and anomalous activities. By extracting actionable insights from traffic data, NIDS can effectively identify potential intrusion threats and trigger timely security alerts, thereby mounting an effective defense against potential cyber-attacks and ensuring the integrity and availability of critical computational resources.

Early network intrusion detection systems learned to identify malicious traffic by combining manually designed features with machine learning models, enabling either signature-based [1] or anomaly-based [2] detection. However, effectively processing and inspecting the massive and dynamic network traffic in the high-bandwidth, low-latency, and burst-intensive scenarios of HPC systems presents a significant challenge. Machine learning-based methods face multiple limitations in the HPC environment due to their over-reliance on handcrafted features. The design of these features is complex and time-consuming, making it difficult to comprehensively capture the diverse and rapidly evolving traffic patterns in HPC systems. This leads to a higher rate of missed detections for novel or variant attacks. Moreover, feature engineering requires extensive domain knowledge, is costly to maintain, and provides insufficient coverage to handle high-dimensional, heterogeneous traffic data.

Deep learning techniques have offered a new direction for the development of Intrusion Detection Systems (IDS). Neural networks can automatically learn hidden features from vast amounts of data, eliminating the need for laborious manual feature engineering and significantly improving the performance of these systems. In recent years, Graph Neural Networks (GNNs) [3–5] have become powerful tools in various fields due to their ability to extract deep structural and relational features from complex data. This capability has sparked widespread interest in modeling network traffic data as graph structures. Network traffic is typically analyzed in the form of flows, which are defined by communication endpoints that include elements such as IP addresses, port numbers, and transport protocols. These flows are further enriched with additional attributes, such as packet byte counts, duration, and other flow-specific metadata. In a graph representation, communication endpoints are modeled as nodes, while the network traffic patterns between them are represented as edges, supplemented with topological and contextual information. The rich information embedded within these edges is crucial for tasks such as network traffic classification and the detection of anomalous traffic patterns. However, in real-world scenarios, network traffic data suffers from a severe imbalance problem, where malicious traffic often constitutes a very small fraction of the data. This leads to poor performance in existing deep learning-based network intrusion detection systems, as these algorithms typically assume a relatively balanced data distribution [6]. Furthermore, data quality issues, such as the high cost of acquiring labeled data, information loss, redundancy, and errors, further exacerbate the difficulty of developing effective anomaly detection models.

To address these challenges, we proposes the Traffic Interpolation Contrastive Learning framework named TICL. Inspired by E-GraphSAGE [7], TICL introduces two innovative components: a data augmentation module and a graph contrastive learning module. The data augmentation module uses enhanced feature interpolation techniques to generate additional samples, mitigating class imbalance in network traffic data. The contrastive learning module improves the discriminative capability of TICL by distinguishing between positive and negative samples. Together, these components enable the TICL framework to achieve state-of-the-art performance in network intrusion detection tasks. The major contributions of our work are summarized as follows:

- **Powerful intrusion detection algorithm:** TICL models network traffic as a graph, effectively capturing complex topologies and communication patterns to enhance the detection of potential intrusions.
- **Effective traffic augmentation technique:** To address class imbalance in HPC network traffic, we propose a novel interpolation-based augmentation method that generates high-quality synthetic samples, improving model robustness and performance.
- **Contrastive learning integration:** A specialized contrastive learning module is designed to generate discriminative feature representations, enabling the model to maintain high detection accuracy even in data-scarce scenarios.
- **State-of-the-art detection performance:** Experimental results show that TICL, which combines data augmentation and contrastive learning, achieves outstanding performance in network intrusion detection tasks.

The remainder of this paper is organized as follows: Sect 2 reviews the related work on network intrusion detection, Graph Neural Networks, and relevant data augmentation techniques. Sect 3 details the architecture and core components of our proposed TICL model. Sect 4 describes the experimental evaluation, including datasets, evaluation metrics, baseline methods and experimental results. Finally, Sect 5 concludes the paper and discusses future research directions.

## 2 Related work

### 2.1 Deep learning approaches for intrusion detection

Deep learning has revolutionized intrusion detection by automating the feature extraction process and improving the accuracy and efficiency of detection systems. Unlike traditional machine learning methods, which rely heavily on manual feature engineering, deep learning models such as Convolutional Neural Networks (CNNs) and Long Short-Term Memory networks (LSTMs) can learn hierarchical representations from raw network data. CNNs are adept at capturing spatial patterns [8], while LSTMs excel in detecting temporal dependencies [9]. Unsupervised deep learning approaches, including Auto-Encoders and Generative Adversarial Networks (GANs), are also widely used in anomaly detection tasks, particularly when labeled data is scarce [10–12]. Despite their strengths, deep learning models face challenges in real-world applications due to the high cost of obtaining labeled data and the dynamic nature of attack patterns, such as Advanced Persistent Threats (APTs) and zero-day attacks. This limitation has driven research into self-supervised and weakly supervised learning paradigms, which leverage unlabeled data to enhance the adaptability and robustness of models.

### 2.2 Graph-based approaches for intrusion detection

In recent years, graph-structured data has gained significant attention in network intrusion detection, owing to its ability to provide a sophisticated abstraction that captures complex interactions among entities such as hosts, IP addresses, and ports. On one hand, graph data encodes local structural information between nodes and their neighbors, enabling models to learn meaningful representations even from unlabeled datasets. On the other hand, graph structures retain semantic and contextual dependencies inherent in network traffic, offering richer feature representations for intrusion detection. Beyond point-to-point relationships, graph data effectively models global structures and localized patterns, thereby enhancing the ability to detect complex attack behaviors.

Graph-based deep learning methods have shown remarkable potential in the cybersecurity domain. For instance, Xu et al. [13] proposed a self-supervised graph embedding approach that enables efficient anomaly detection in network traffic, even in scenarios with limited labeled data. Similarly, Lin et al. [14] developed E-GRACL, an IoT-targeted intrusion detection system leveraging Graph Neural Networks (GNNs) and self-supervised learning to identify zero-day attacks. These methods improve model robustness against noise while enhancing generalization to novel attack patterns. Overall, graph-based learning approaches effectively model complex network interactions, reduce reliance on labeled data, and provide innovative solutions to challenges in network intrusion detection.

In addition, contrastive learning based on graph-structured data has achieved notable progress in addressing the scarcity of labeled data for intrusion detection. Contrastive learning constructs positive and negative sample pairs, allowing models to uncover latent attack patterns and improve classification performance. For example, Farrukh et al. [15] introduced a dual-modal framework called XG-NID, which combines Heterogeneous Graph Neural Networks (HGNNs) and Large Language Models (LLMs) to achieve real-time intrusion detection with high interpretability by integrating flow-level and packet-level data. Similarly, Hu et al. [16] developed an early intrusion detection method based on graph embeddings, where flow graphs are constructed from initial network packets and classified using random forests, resulting in significant improvements in accuracy and efficiency. These studies highlight the immense potential of graph-structured and contrastive learning approaches in addressing data scarcity and detecting complex attack patterns, offering innovative solutions for modern network intrusion detection challenges.

### 2.3 Self-supervised and weakly-supervised learning paradigms

In the field of weakly-supervised learning, researchers are dedicated to developing techniques that leverage small amounts of labeled data or partial annotations to address the challenge of scarce labeled data in network intrusion detection. Weakly-supervised learning combines unsupervised, semi-supervised, and self-supervised learning techniques to uncover latent attack patterns from unlabeled data, thereby significantly reducing reliance on high-quality labeled data. For example, Li et al. [17] proposed a graph meta-learning framework, GMFITD, for few-shot insider threat detection. By integrating graph model auto-encoders with attention mechanisms, GMFITD effectively captures potential relationships among users and achieves efficient anomaly detection in few-shot scenarios. Additionally, generative models such as GANs have been used to synthesize data, enhancing model generalization in weakly-supervised settings. For instance, Zhang et al. [18] developed a GAN-based synthetic anomaly generation method, which generates realistic anomaly data to improve model generalization, enabling efficient intrusion detection even with limited labeled data.

Contrastive learning, as a self-supervised learning technique, plays a pivotal role in weakly-supervised learning. Its core idea is to construct positive and negative sample pairs, enabling models to learn meaningful representations from unlabeled data. In graph data, contrastive learning methods capture local structural information between nodes and their neighbors, effectively uncovering latent attack patterns in network traffic. For example, Duan et al. [19] proposed a multi-view contrastive learning method for graph anomaly detection, which constructs multi-view contrastive objectives based on network structure, node evolution, and topology evolution, significantly improving anomaly detection performance in dynamic networks. This method not only captures local dependencies among nodes but also enhances model performance under low-quality pseudo-labels by introducing global distribution prototypes and virtual negative samples.

Self-supervised contrastive learning methods for anomaly detection, such as Anemone, have achieved notable progress. This method constructs contrastive sample pairs between nodes and their local substructures, learning meaningful representations from unlabeled data and significantly improving anomaly detection performance [20]. Anemone not only reduces reliance on labeled data but also mitigates the impact of noise by learning local connectivity information in attributed networks, while reducing computational complexity by avoiding direct input of the entire network into the model. Furthermore, Liu et al. [21] proposed a real-time intrusion detection method based on spatio-temporal graph neural networks, which captures spatio-temporal dependencies in network traffic, significantly enhancing detection accuracy. These studies demonstrate the immense potential of weakly-supervised learning in network intrusion detection, effectively addressing the challenge of scarce labeled data and significantly improving model detection performance.

## 3 Methodology

**Fig 1:**
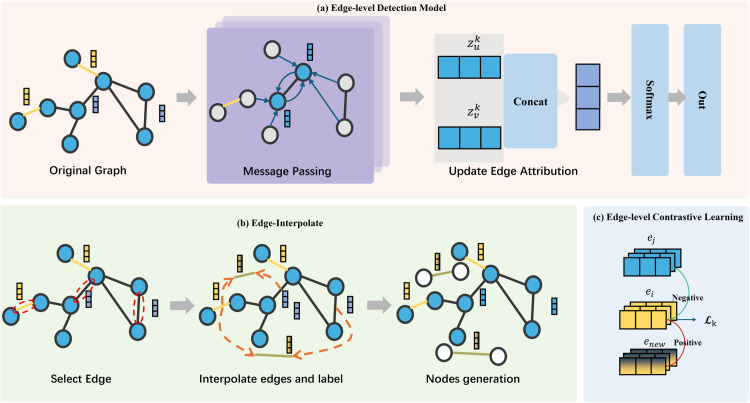
The architecture of TICL. The complete workflow proceeds as follows: 1) Sample edges in the original graph and perform edge interpolation; 2) Using augmented data for contrastive learning; 3) Input the original graph into the GNN model to update the edge attributes; 4) Combining contrastive learning with the edge information learned by the GNN model for traffic detection.

The proposed TICL model in this paper consists of three main components: 1) A data augmentation module based on edge interpolation; 2) A feature representation module based on contrastive learning; 3) A traffic detection module based on graph neural networks. The overall framework of the model is illustrated in the Fig 1.

### 3.1 Traffic graph construction

Network traffic data inherently exhibits graph-structured properties, where the utilization of graph structures can naturally capture topological relationships and interaction patterns within the traffic. Moreover, network traffic data typically encompasses rich contextual information, such as traffic direction, protocol type, and packet size. These features provide multidimensional inputs for GNNs, enabling them to effectively capture both local and global traffic patterns, thereby facilitating the identification of potential anomalous behaviors. Based on this rationale, this study models network traffic data as graph structures to leverage the capabilities of GNNs for intrusion detection tasks.

In the intrusion detection task of this study, we first extract five-tuple information (source IP, source port, destination IP, destination port, and protocol) from the network traffic data. The binary tuples (source IP and source port, or destination IP and destination port) are then defined as nodes in the network traffic graph. Through this approach, devices in the network are mapped to nodes in the graph, while the edges between source and destination nodes represent the network traffic transmitted between these devices.

The coarse-grained modeling strategy of defining nodes as binary tuples is a key design choice aimed at addressing the core challenges of scalability and efficiency when applying GNNs to real-world network traffic data. Primarily, when dealing with massive volumes of traffic data, finer-grained approaches—such as modeling individual network flows as nodes—would result in a graph scale that becomes computationally infeasible, severely limiting the model’s value for practical deployment. More importantly, this strategy is highly synergistic with the core of our framework, which is edge-level contrastive learning. By simplifying nodes to represent communication endpoints, the model’s learning focus is guided towards the traffic interactions (i.e., edges) that form the core of the analysis. This approach enhances learning efficiency while maintaining a manageable computational overhead. The effectiveness of this design choice was also empirically validated through our preliminary experiments.

To mitigate potential biases of the model towards specific IP addresses, this study applies a mapping process to both source and destination IP addresses. Specifically, source IP addresses are mapped to the private address range 10.0.0.0~10.255.255.255, while destination IP addresses are mapped to the private address range 192.168.0.0~192.168.255.255. Additionally, considering that the message-passing mechanism of GNNs may be susceptible to noise interference from node features, this study initializes the feature values of all nodes to 0, indicating the absence of any feature information. This design aims to ensure that the model focuses more effectively on edge detection tasks, thereby improving the accuracy of detection results.

### 3.2 Traffic interpolation contrastive learning

#### 3.2.1 Traffic interpolation module.

In real-world network scenarios, traffic data often exhibits significant imbalance, which can lead to degraded model performance during training. Existing methods fail to adequately address this imbalance. To tackle this issue, we propose an edge-based data interpolation method for data augmentation, specifically designed to mitigate data imbalance in real-world settings. Traditional graph-based interpolation methods typically generate new samples through linear combinations, which are only suitable for node-level classification tasks and are not applicable to our edge classification scenario. Therefore, our approach introduces an interpolation method constrained by the positive-to-negative sample ratio α, where edges are sampled from the original data for interpolation-based augmentation. Specifically, in TICL, we construct a sample pool from the edges labeled as positive (malicious) and negative (benign) in the original traffic. For each interpolation, a positive edge *e*_*i*_ and a negative edge *e*_*j*_ are randomly drawn from this pool. A new edge feature, *e*_*new*_, is then generated according to the interpolation coefficient β using the formula: $e_{new}=\beta \cdot e_{i}\;+\;\left(1\;-\;\beta \right)\cdot e_{j}$. If β>0.5, the new edge is assigned a positive label; otherwise, it is assigned a negative label. Throughout this process, the ratio of sampled negative to positive samples is maintained at α. This allows the minority class to be augmented on-the-fly without requiring any additional labeling, thereby mitigating the class imbalance problem.

Here, β∈[0,1] represents the interpolation coefficient, which controls the weight allocation between the features of the two edges. After implementing TICL, it is necessary to annotate the newly generated edge data. To achieve this, we introduce an indicator function to label the augmented data. Specifically, the labeling is controlled by the interpolation coefficient. When λ>0.5, the indicator function assigns a label of 0 (indicating a benign edge); when λ≤0.5, the edge is labeled as 1 (indicating a malicious edge). This labeling strategy effectively captures the categorical tendency of the interpolated edges, thereby providing more accurate supervisory signals for the model. The indicator function is formally defined in Eq 1.

$$\hat{y}=𝕀(0<\lambda <0.5)$$ (1)

#### 3.2.2 Contrastive learning module.

To fully leverage the extensive augmented data generated by TICL, we introduces a contrastive learning-based feature learning strategy, aiming to enhance the model’s understanding of data distributions. This approach constructs supervisory signals by utilizing the inherent structural information of the data, thereby uncovering latent relationships among samples to optimize the model training process. Within the framework of this study, we employ the InfoNCE loss function [22] to extract feature representations from the virtual samples generated by TICL. These representations are then combined with positive samples from the original dataset to form new positive sample pairs. Simultaneously, negative samples are randomly selected from the dataset, and the similarity between positive and negative samples is computed to refine the feature space. Throughout this process, the ratio of positive to negative samples is set to γ. The InfoNCE loss function is defined as follows:

$${ℒ}_{k}=-\frac{1}{N}\sum_{e^{\text{Pos}}\in {ℰ}^{\text{Pos}}}\log(\frac{\exp(e^{\text{Pos}}\cdot e_{i})}{\exp(e^{\text{Pos}}\cdot e_{i})+\sum_{e^{\text{Neg}}\in ℰ}\exp(e^{\text{Neg}}\cdot e_{i})})$$ (2)

Where *e*_*i*_ represents a positive sample edge in the edge set ℰ, ${ℰ}^{\text{Pos}}$ denotes the set of positive samples within ℰ (with $e^{\text{Pos}}\in {ℰ}^{\text{Pos}}$), and ${ℰ}^{\text{Neg}}$ represents the set of negative samples within ℰ (with $e^{\text{Neg}}\in {ℰ}^{\text{Neg}}$).

### 3.3 Traffic detection model

Conventional applications of GNNs have largely concentrated on tasks related to node classification, with relatively little attention given to edge classification problems. To address this gap, this work introduces a novel edge classification model inspired by the E-GraphSAGE algorithm [7]. The model is designed to incorporate edge information into node embeddings through GNN-based mechanisms, such as message passing and neighborhood aggregation. By doing so, it enables effective modeling and classification of edge-level features. The model achieves this by aggregating information from both the source and target nodes of an edge, as well as their respective neighborhoods, and incorporating edge-specific attributes. This aggregation process, which forms the basis of the edge embedding generation, is mathematically described as:

$${𝐡}_{𝒩(v)}^{k}\leftarrow {Aggregation}_{k}\left(\left\{{𝐡}_{u}^{k-1}\;\|\;{𝐞}_{uv}^{k-1},\forall u\in 𝒩(v),uv\in ℰ\right\}\right)$$ (3)

where ${𝐞}_{uv}^{k-1}$ refer to the feature vector of edges between nodes *u* and *v* in the (*k*–1)-th layer, and 𝒩(v) denotes the set of nodes connected to *v*. The aggregation process integrates edge features with node embeddings to iteratively update representations. At each layer *k*, node embeddings incorporate structural and contextual information from their *k*-hop neighbors, while edge embeddings are derived from these updated node representations. This iterative refinement ensures that the model captures both local and global structural patterns in the graph.

The representation of a node at the *K*-th layer is finalized as ${𝐳}_{v}={𝐡}_{v}^{K}$, encapsulating all aggregated information up to that layer. For each edge, the final embedding ${𝐳}_{u\nu }^{K}$ is derived by concatenating the embeddings of its associated nodes of *u* and *v*, as illustrated in Eq 4.

$${𝐳}_{u\nu }^{K}\leftarrow Concat\left({𝐳}_{u}^{K},{𝐳}_{\nu }^{K}\right),u\nu \in ℰ$$ (4)

### 3.4 Model train

To train the proposed model, the primary objective is to minimize the cross-entropy loss, which serves as the core classification loss function. The cross-entropy loss is computed as follows:

$${ℒ}_{c}=\frac{1}{N}\sum_{i}-[y_{i}\cdot \log(p_{i})+(1-y_{i})\cdot \log(1-p_{i})]$$ (5)

Here, *y*_*i*_ represents the true class label of the *i*-th sample, with 0 representing a positive instance and 1 indicating a negative instance. Additionally, *p*_*i*_ refers to the predicted probability of the *i*-th sample being classified as positive.

The overall training objective integrates the cross-entropy loss ${ℒ}_{\text{c}}$ with an additional contrastive loss ${ℒ}_{\text{k}}$, which enhances the model’s capability to learn meaningful representations. The combined loss function is expressed as:

$$ℒ={ℒ}_{\text{c}}\;+\theta {ℒ}_{\text{k}}$$ (6)

Among them, θ is an learnable parameter.

## 4 Experiments

To assess the effectiveness of the TICL, we conducted experiments focused on network intrusion detection using two public datasets. Sect 4.1 introduces the datasets, while Sect 4.2 provides an overview of the baseline methods used for comparison. Sect 4.3 details the experimental design, including steps for data preparation and parameter configurations. The evaluation metrics employed to measure the model’s performance are discussed in Sect 4.4. In Sect 4.5, we analyze the model’s performance on intrusion detection tasks and compare it against established baseline approaches. To further demonstrate the impact of contrastive learning and data augmentation strategies, we carried out ablation studies. Additionally, in Sect 4.6, we explored the influence of varying the number of interpolated samples on model performance by modifying the interpolation parameters.

### 4.1 Data preparation

To demonstrate the effectiveness of TICL for network intrusion detection, we performed experiments using two publicly available network traffic datasets. A summary of the datasets is provided in Table 1.

**Table 1:** Summary of dataset properties.

Dataset	Num.nodes	Num.edges	Features.dim	Num.postive	Num.negtive
BoT-IoT	3,683,084	3,668,522	55	3,682,607	477
UNSW-NB15	1,526,205	1,270,022	33	1,109,367	160,655

**BoT-IoT [23]:** This dataset was created by Koroniotis et al. in 2019 and is a publicly available dataset specifically designed for network attack detection in Internet of Things (IoT) environments. It contains various types of network traffic data, including normal traffic and six different types of attack traffic (e.g., DDoS attacks, scanning attacks, malware propagation, etc.). Koroniotis et al. also extracted 47 traffic features from the raw data using the Argus tool and provided detailed annotations for each traffic sample. The dataset consists of a total of 3,668,045 network traffic records, with malicious traffic accounting for 99.99% and benign traffic only 0.01%.

**UNSW-NB15 [24]:** This dataset was released by Moustafa and Slay in 2015 and is a comprehensive dataset for network intrusion detection systems. It was created by the UNSW Canberra Cyber Range Lab using the IXIA PerfectStorm tool to generate a hybrid of real modern normal traffic and nine types of synthetic attack traffic (e.g., Fuzzers, Backdoors, Exploits, etc.). The authors used the Argus and Bro-IDS tools to extract a total of 49 features from the raw traffic and applied class labels. The dataset contains a total of 2,540,044 records, a portion of which is partitioned into a training set (175,341 records) and a testing set (82,332 records).

### 4.2 Comparative methods

To validate the performance of our model, we compare TICL with the following two commonly used intrusion detection methods.

**E-GraphSAGE:** This method is a graph neural network algorithm based on GraphSAGE, which primarily learns node representations by aggregating neighborhood information of nodes. Additionally, it considers edge features and generates edge embeddings by sampling and aggregating edge information in the graph. This enables E-GraphSAGE to effectively capture local structural information in graph data, thereby achieving network intrusion detection. However, the original E-GraphSAGE algorithm does not incorporate data augmentation or contrastive learning strategies, leading to certain limitations in scenarios with class imbalance, particularly in recognizing minority classes.

**E-GraphSAGE-Res [25]:** E-GraphSAGE-Res introduces residual connections on top of E-GraphSAGE to enhance the information propagation capability of the network and avoid training difficulties caused by information loss or gradient vanishing. At the same time, residual connections provide more training signals for minority classes, thereby improving the overall detection performance of the model in class-imbalanced tasks. In this way, E-GraphSAGE-Res can significantly enhance the classification accuracy of minority class samples while maintaining the complexity and expressive power of the network structure.

### 4.3 Experimental setup

#### 4.3.1 Data preparation.

Prior to the experiments, we standardized the dataset by partitioning it into training, validation, and test sets with a ratio of 7:1:2. To ensure the reliability of the experiments, we balanced the positive and negative samples in each subset during the partitioning process, maintaining an identical ratio of positive to negative instances across all subsets. To further evaluate the robustness of the model, we implemented a five-repeated ten-fold cross-validation strategy and calculated the macro-average F1 score along with its standard deviation based on this approach. This method not only enhances the reliability of the experimental results but also significantly reduces potential biases caused by uneven dataset distribution. Additionally, to ensure fairness in experimental comparisons, the performance evaluation of all models was conducted on a unified test set, thereby guaranteeing the consistency and objectivity of the evaluation results.

#### 4.3.2 Parameter settings.

The TICL model proposed in this paper is implemented based on the PyTorch deep learning framework. The model employs a dual-layer E-GraphSAGE architecture as its core structure, with the embedding dimension of each layer set to 128. To enhance the model’s generalization capability and effectively prevent overfitting, we introduced a dropout mechanism between the two E-GraphSAGE layers, with the dropout rate set to 0.2. During the model training process, we integrated data augmentation strategies and contrastive learning strategies to further improve the model’s learning ability and generalization performance.

In terms of data augmentation, we proposed an innovative strategy based on interpolation. Specifically, this strategy controls the sample mixing process through two key parameters: α is used to adjust the mixing weight coefficient between positive and negative samples, with its value set to 0.3; β is used to control the mixing weight coefficient among positive samples, with its value set to 0.2. Additionally, the parameter σ is used to specify the number of positive and negative samples sampled during the mixing process, with its value fixed at 200. In the contrastive learning setup, we introduced the parameter γ to control the sampling ratio of positive and negative samples, with its value set to 10 to ensure a balanced sample distribution.

The model adopts ReLU (Rectified Linear Unit) as the nonlinear activation function and constructs an output layer classifier through the Softmax function to support the execution of downstream tasks. In terms of optimization strategy, we use the Adam optimizer for parameter updates, with the learning rate set to 0.01 to ensure efficient gradient updates during the backpropagation process. Furthermore, we proposed a composite loss function that combines Cross-Entropy Loss with InfoNCE Loss, thereby simultaneously optimizing the accuracy of classification tasks and the effectiveness of representation learning during training. This design significantly enhances the model’s performance in multi-task learning scenarios.

### 4.4 Model evaluation metrics

To comprehensively assess the performance of the model, we used the Macro F1-Score as the primary evaluation metric. The Macro F1-score is the average of the F1-scores for each class, providing a balanced view of the model’s performance, particularly in cases of class imbalance. The F1 score is a widely used metric in binary classification tasks that balances precision and recall, and its calculation is given by:

$$F1=2\times \frac{Precision\times Recall}{Precision+Recall},$$ (7)

$$\text{ Macro F1 }=\frac{1}{N}\sum_{i=1}^{N}F1_{i}$$ (8)

Here, precision indicates the model’s ability to correctly identify positive instances, while recall reflects the model’s capability to capture all actual positive instances. A higher F1-score implies a better balance between precision and recall, thus indicating better overall performance. The Macro F1-score is particularly useful in multi-class classification tasks. It computes the F1-score for each class individually and averages them. This approach ensures that the model’s performance is fairly evaluated across all classes, reducing the influence of dominant classes and offering a more holistic view of its effectiveness.

### 4.5 Experimental results

We compare the proposed TICL model with commonly used intrusion detection methods, including E-GraphSAGE, E-GraphSAGE-Res, and E-ResGAT, by evaluating their macro-average F1-scores on the BoT-IoT and UNSW-NB15 datasets. The experimental results, presented in Table 2, clearly show that TICL outperforms all other methods on both datasets. Specifically, on the BoT-IoT dataset, TICL achieves a macro-average F1-score of 95.43%, surpassing the second-best method by 0.49%. On the UNSW-NB15 dataset, TICL achieves a macro-average F1-score of 97.77%, which is 0.03% higher than the second-best method.

**Table 2:** Performance of the intrusion detection task for different training set split ratios, expressed as macro-f1 and standard deviation.

Method	Train Split 60%		Train Split 40%	
	Bot-IoT	UNSW-NB15	BoT-IoT	UNSW-NB15
E-GraphSAGE	94.03 ± 1.12	97.68 ± 0.07	94.94 ± 1.88	97.66 ± 0.03
E-GraphSAGE-Res	94.03 ± 1.12	97.69 ± 0.01	94.94 ± 1.88	97.74 ± 0.02
E-ResGAT	54.70 ± 19.26	44.75 ± 45	48.76 ± 0.65	40.77 ± 51
TICL-CL	94.03 ± 1.12	97.76 ± 0.03	95.14 ± 2.07	97.72 ± 0.02
TICL-EI	92.87 ± 1.20	97.69 ± 0.03	92.43 ± 0.63	97.73 ± 0.01
TICL	**95.28 ± 0.44**	**97.80 ± 0.03**	**95.43 ± 0.63**	**97.77 ± 0.03**

Additionally, to assess the influence of data augmentation and contrastive learning on model performance, we conducted ablation experiments. The results, shown in Table 2, reveal that both strategies—edge interpolation-based data augmentation and contrastive learning—offer improvements over the baseline methods on both datasets. These findings emphasize the effectiveness of combining these two strategies. The edge representations derived from edge interpolation and contrastive learning are essential for detecting anomalous edges, thereby boosting the model’s overall performance.

**Fig 2:**
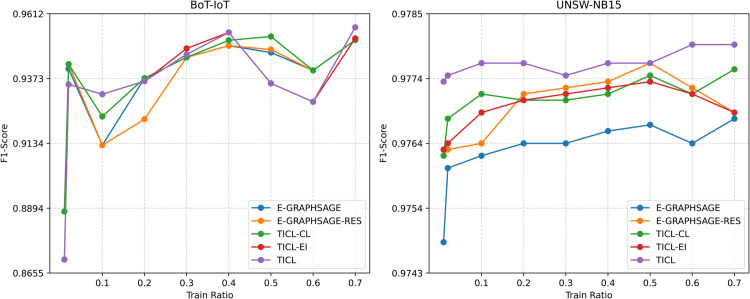
Performance comparison of different methods under various data partitioning schemes.

To further highlight the superiority of the TICL model, we performed data partitioning experiments, which demonstrate the advantages of integrating contrastive learning and data augmentation for intrusion detection. As illustrated in Fig 2, the performance of various methods was compared across different training set sizes (ranging from 1% to 60% of the total dataset). The results indicate that TICL, along with its TICL-CL and TICL-EI variants, consistently outperforms the baseline models on both datasets. These experiments validate the effectiveness of our proposed approach. Moreover, we observed that on the BoT-IoT dataset, as the training set size increases, the performance of all models exhibits fluctuations. However, TICL maintains a relatively stable performance, with smaller fluctuations compared to the baseline models, and consistently achieves the highest macro-average F1-score.

Furthermore, to evaluate the robustness of our proposed TICL method, we conducted migration experiments. Specifically, we trained and tested TICL on the BoT-IoT and UNSW-NB15 datasets. The experimental results are shown in Table 3. As can be seen, the TICL method outperforms the baseline method in both model performance and robustness. This is primarily attributed to the contrastive learning module, which enhances cross-domain consistency of edge representations through self-supervised learning. Specifically, in the migration scenario, the improvement in F1 stems from the contrastive learning’s improved negative sampling and similarity optimization of minority edges, while the reduction in ↓ reflects the model’s tolerance to distribution drift. Further ablation analysis shows that removing the contrastive learning component increases ↓ by 10% and 15% on the two datasets, respectively, while retaining the self-supervised loss only reduces performance by 5% and 2%, confirming the dominant role of contrastive learning in robustness.

**Table 3:** Performance of the intrusion detection task in the migration scenario, expressed as macro f1 and standard deviation.

	BoT to UNSW			UNSW to BoT		
	F1	std	↓	F1	std	↓
E-GraphSAGE	84.13	1.18	11.02	61.85	21.53	35.83
E-GraphSAGE-Res	80.26	1.25	14.89	72.86	18.55	24.83
TICL-CL	89.12	2.78	6.03	93.55	0.04	4.21
TICL-EI	84.98	3.55	10.23	82.35	21.66	15.34
TICL	**90.24**	**0.8**	**5.38**	**95.63**	**1.27**	**2.17**

Collectively, the experimental results consistently demonstrate the outstanding performance and robustness of the TICL framework. This performance enhancement is primarily attributed to the synergy between its two core components: the interpolation-based data augmentation strategy effectively mitigates the class imbalance problem at the data level, while the contrastive learning module strengthens the model’s discriminative capabilities at the feature level. These findings have twofold implications. Theoretically, this work provides an effective paradigm for applying GNNs to imbalanced cybersecurity datasets, underscoring the importance of combining advanced architectures with specialized data and representation learning strategies. Practically, the robust performance of TICL, even with limited training data, highlights its significant potential for deployment in next-generation Network Intrusion Detection Systems to better protect critical HPC infrastructure.

### 4.6 Model parameter analysis

**Fig 3:**
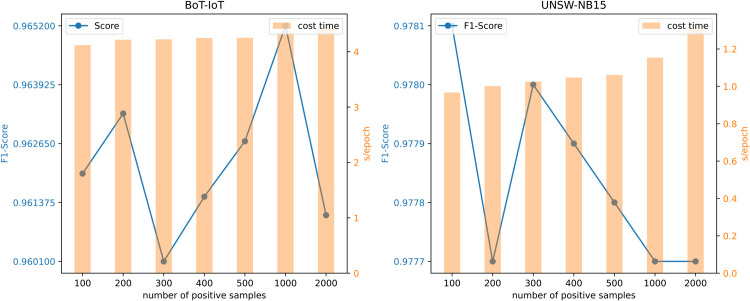
Impact of different sampling sizes in the interpolation method on model performance.

In the proposed approach, we integrated the interpolation data augmentation strategy to enhance the diversity of the training samples by combining existing data samples, ultimately improving the model’s generalization ability. To optimize this data augmentation strategy, we conducted parameter experiments to determine the ideal number of samples for the interpolation method. The experimental results are illustrated in Fig 3. Specifically, we evaluated the model’s performance using varying numbers of interpolation samples, ranging from 100 to 2000, to identify the optimal number of samples.

The results for the BoT-IoT dataset, which exhibits severe class imbalance, indicate that a larger number of interpolated samples generally leads to better performance. As shown in the figure, while some fluctuations are present, the overall trend demonstrates that more augmentation helps the model overcome the data scarcity of the minority attack class. To achieve a significant performance gain without incurring excessive computational costs, we selected 1000 samples as the optimal number for the BoT-IoT dataset.

In contrast, the UNSW-NB15 dataset, which is relatively balanced, demonstrated that the model’s performance peaked at 100 samples and gradually declined as the number of samples increased beyond this threshold. We hypothesize that for more balanced datasets, excessive data augmentation is not required and can even negatively impact performance by distorting the natural data distribution. Therefore, we selected 100 samples as the optimal number for the UNSW-NB15 dataset.

Based on these findings, we conclude that the optimal number of interpolation samples is highly dependent on the specific class distribution of the dataset. Consequently, our final model configuration uses 1000 samples for the severely imbalanced BoT-IoT dataset and 100 samples for the more balanced UNSW-NB15 dataset to maximize performance while considering computational efficiency.

### 4.7 Graph construction analysis

**Fig 4:**
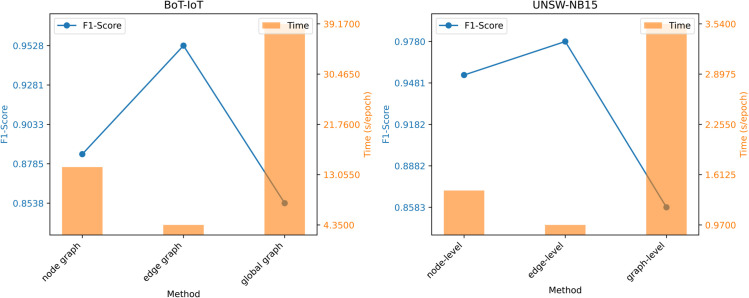
Impact of different graph construction method on model performance.

To verify the superiority of the proposed graph construction method based on binary tuples, this paper systematically compares three typical graph construction strategies: (1) traffic as nodes (node classification task); (2) traffic as edges (our method); and (3) traffic packets as nodes (graph classification task). Experiments are conducted on the BoT-IoT and UNSW-NB15 datasets, with all model parameters kept consistent. Evaluation metrics include F1 score and training efficiency. The experimental results are shown in Fig 4. The proposed edge classification method with traffic as edges significantly outperforms the other two strategies in F1 score. This is mainly attributed to the high-dimensional feature representation of binary tuples, which can capture richer traffic interaction information rather than a local view of isolated packets. At the same time, this method uses binary tuplesas nodes, which effectively suppresses the expansion of node scale, thereby achieving better computational efficiency in large-scale network traffic scenarios. These results further confirm the advantages of the TICL framework in robustness and scalability.

### 4.8 Efficiency analysis

To evaluate the impact of the interpolation augmentation and contrastive learning modules on the computational overhead of the proposed TICL framework, this paper systematically assesses its computational efficiency from both theoretical and experimental perspectives.

Theoretically, assuming the training graph contains *N* nodes, *E* edges, a feature dimension of *d*, *H* minority-class (malicious) samples, and an *L*-layer GNN, the time complexity for each forward propagation layer is O(E·d), the full forward-backward propagation complexity is O(L·E·d), and the space complexity is O(E·d+N·d). The interpolation augmentation generates $O(H\cdot U^{\prime })$ additional edges through subsampling (where $U^{\prime }$ is the size of a fixed benign subset), linearly expanding the number of edges to $E^{\prime }=E+O(H\cdot U^{\prime })$. This increases the total training complexity to $O(T\cdot L\cdot E^{\prime }\cdot d)$, with an increment of $O(T\cdot L\cdot H\cdot U^{\prime }\cdot d)$ (where *T* is the number of epochs). Contrastive learning adds an overhead of O(B·d·K) per batch (where *B* is the batch size and *K* is the fixed negative sampling ratio), resulting in a total training increment of O(T·B·d·K). This demonstrates that the introduction of the interpolation augmentation and contrastive learning modules only linearly scales the complexity of the baseline model, without causing a higher-order complexity explosion.

**Fig 5:**
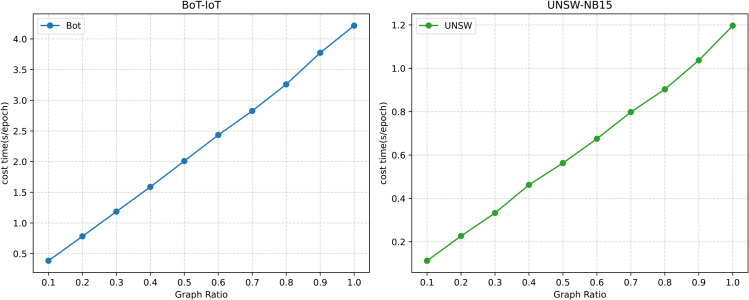
Impact of different graph ratio on model performance.

To validate this theoretical analysis, Fig 3 and Fig 5 respectively show the training efficiency curves under varying sampling quantities and graph scales. The results show that the model’s training time has a positive linear correlation with the sampling quantity and graph scale, further confirming the computational scalability of the method.

## 5 Conclusions

In the domain of intrusion detection for HPC systems, the application of Graph Neural Networks (GNNs) remains exploratory. This paper proposed the TICL algorithm, which effectively addresses data imbalance in real-world network traffic through innovative data interpolation and contrastive learning. Experimental evaluations demonstrated that the TICL framework outperforms existing methods, showing significant potential for enhancing HPC security. These findings not only provide academics with an effective paradigm for applying GNNs to highly imbalanced cybersecurity data but also offer a technical blueprint for enterprises and policymakers to develop next-generation intelligent intrusion detection systems. Despite these promising results, the study has limitations; for instance, its effectiveness was primarily validated on specific datasets, and the large-scale data augmentation introduces additional computational overhead. Therefore, future research should focus on testing the framework’s generalizability in more diverse network environments, exploring more efficient model optimization techniques to reduce computational costs, and investigating advanced self-supervised learning methods to further decrease reliance on labeled data.
